# Supplementary figures and images for: Variations in bacterial diversity and community structure in the sediments of an alkaline lake in Inner Mongolia plateau, China
Source: PeerJ. 2023 Aug 21;11:e15909. doi: 10.7717/peerj.15909 (PMC10448878; doi:10.7717/peerj.15909)

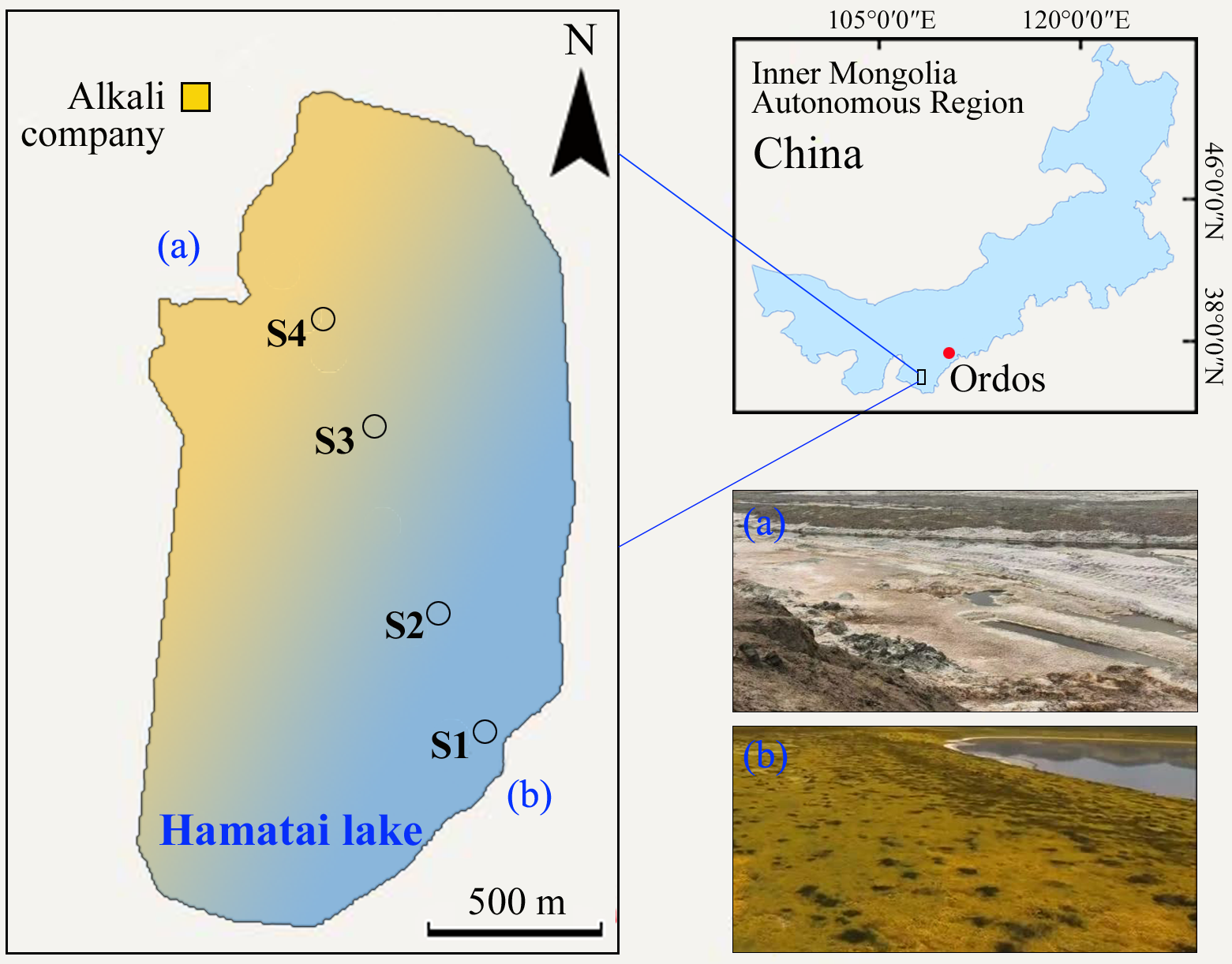

Supplement: Supplemental Information 1 [file peerj-11-15909-s001.png]

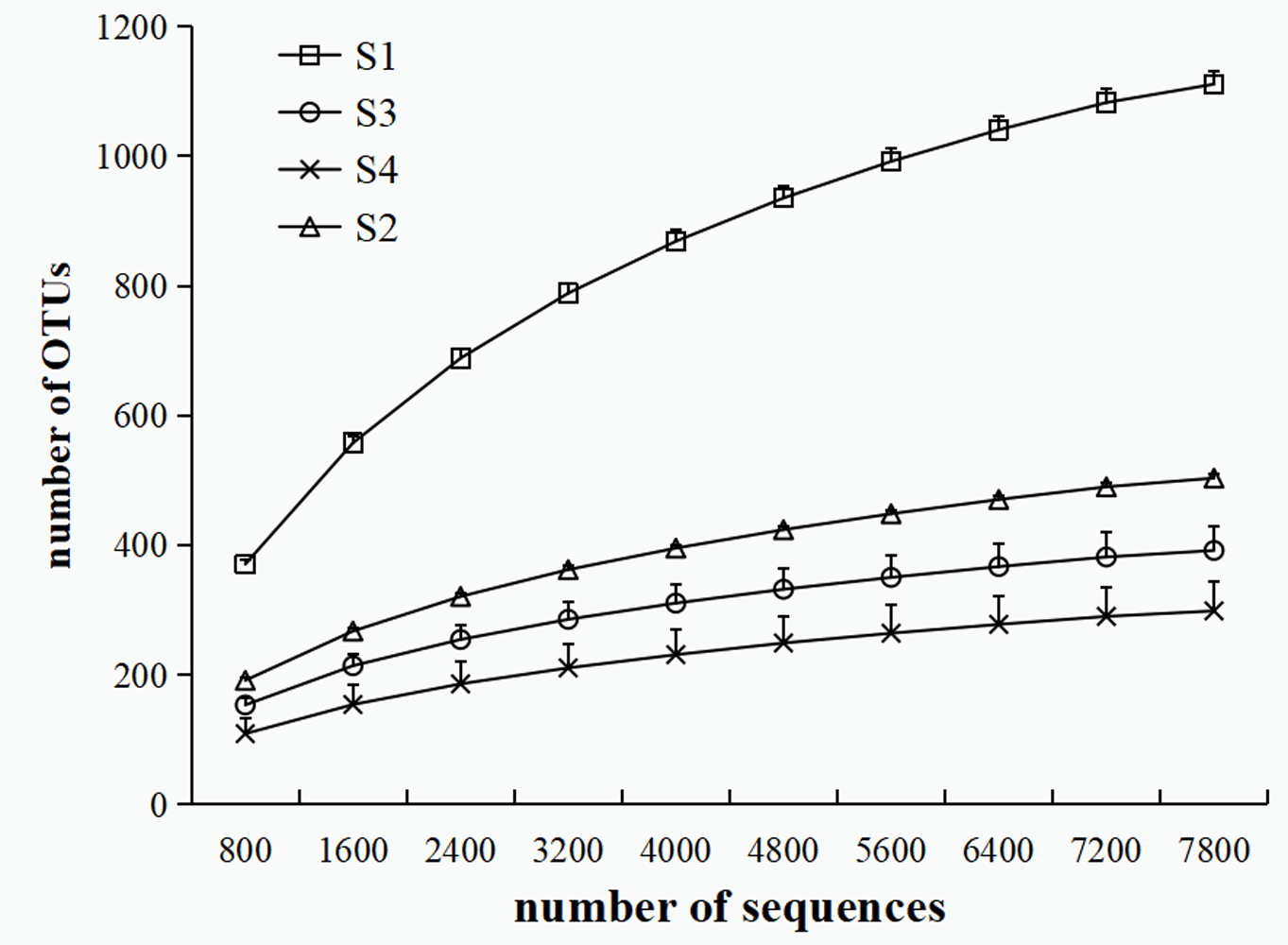

Supplement: Supplemental Information 2 [file peerj-11-15909-s002.png]

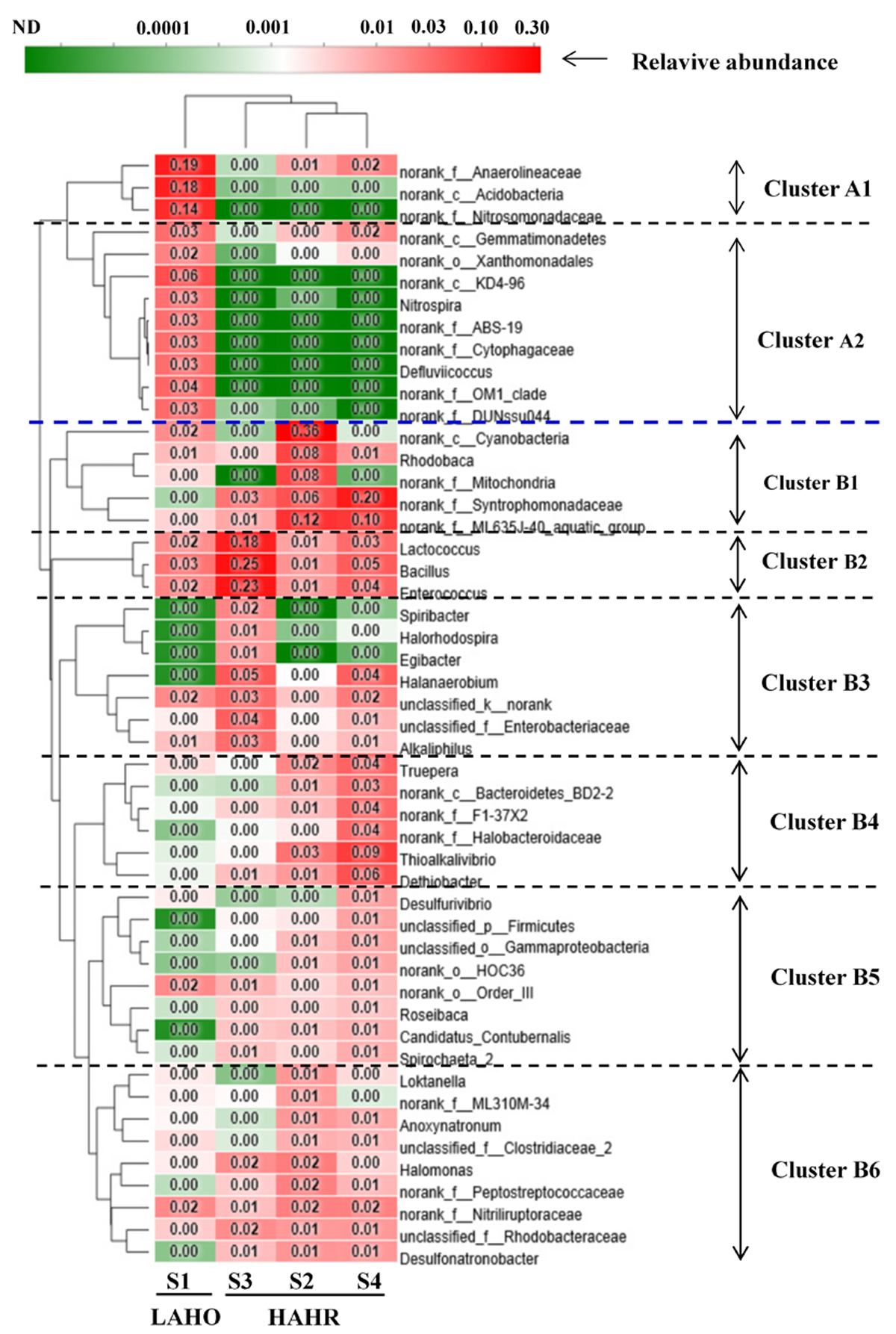

Supplement: Supplemental Information 3 — The color intensity and the number in each cell both shows the relative percentage of a taxa at fine level in a sample. LAHO: relatively light alkaline and hyposaline sediment; and HAHR: high alkaline and hypersaline sediment. [file peerj-11-15909-s003.png]

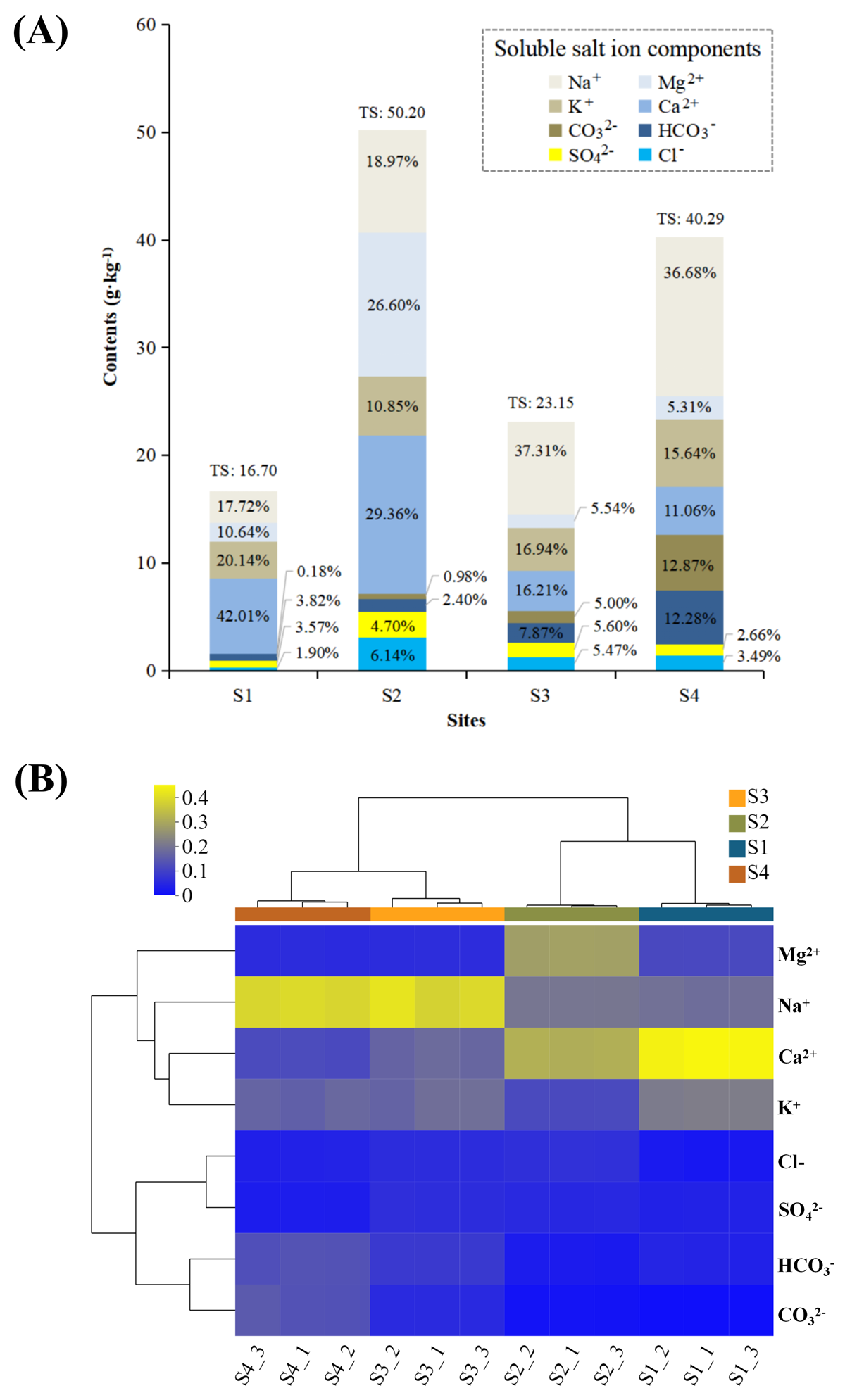

Supplement: Supplemental Information 4 — Cl− and SO42− concentration were determined by Ion Chromatograph (ICS-900, DIONEX, USA). CO32− and HCO3− concentration were detected using the chemical titration method. Total concentration of the K+, Ca2+, Na+ and Mg2+ were measured with an inductively coupled plasma–optical emission spectrometer (ICP–OES, iCAP6000, Thermo Fisher Scientific, USA) (Xiong et al., 2012). TS (sediment total salinity) was characterized by calculating the sum of soluble salt ion components (Zhang et al., 2021b). [file peerj-11-15909-s004.png]
